# Insulin like growth factor 2 mRNA binding protein 2 regulates vascular development in cerebral arteriovenous malformations

**DOI:** 10.3389/fneur.2024.1483016

**Published:** 2024-12-11

**Authors:** Lin-jian Wang, Yangyang Wu, Sha Xie, Hongkai Lian

**Affiliations:** ^1^Department of Neurosurgery, Zhengzhou Central Hospital Affiliated to Zhengzhou University, Zhengzhou, China; ^2^Institute of Trauma and Metabolism, Zhengzhou University, Zhengzhou, China; ^3^Tianjian Laboratory of Advanced Biomedical Sciences, Zhengzhou, China; ^4^School of Medicine, Henan University of Chinese Medicine, Zhengzhou, China

**Keywords:** cerebral AVMs, IGF2BP2, m^6^A, LGALS8, cerebrovascular dysplasia

## Abstract

**Background:**

Cerebral arteriovenous malformations (AVMs) are intricate vascular anomalies that disrupt normal cerebral blood flow, potentially leading to severe neurological complications. Although the pathology of AVMs is not fully understood, epigenetic mechanisms have been implicated in their formation.

**Methods:**

Transcriptional differences between cerebral AVMs and normal tissues were analyzed using RNA sequencing (RNA-seq), identifying IGF2BP2 as a key differentially expressed gene. Comprehensive bioinformatics analysis, integrating multi-omics data such as RNA-seq and methylated RNA immunoprecipitation sequencing (MeRIP-seq), was employed to identify the downstream target gene of IGF2BP2. The roles of specific genes in vascular development were assessed using endothelial cell cultures and zebrafish models.

**Results:**

Our analysis of RNA-seq data from cerebral AVMs and normal tissues identified IGF2BP2, a key N^6^-methyladenosine (m^6^A) reader, as significantly downregulated in cerebral AVMs. Functional studies showed that IGF2BP2 knockdown resulted in abnormal angiogenesis in endothelial cells and disrupted vascular development in zebrafish models. Mechanistically, IGF2BP2 regulates LGALS8 expression by modulating mRNA stability through m^6^A modification, and LGALS8 deficiency severely impairs angiogenesis *in vitro* and leads to cerebrovascular dysplasia *in vivo*.

**Conclusion:**

Our findings suggest that IGF2BP2, via m^6^A-dependent regulation of LGALS8, is crucial for vascular development and presents potential targets for therapeutic intervention in cerebral AVMs.

## Graphical abstract

IGF2BP2 is significantly downregulated in cerebral arteriovenous malformations (AVMs), reducing LGALS8 mRNA stability through m6A modification, thereby severely impairing brain vascular development *in vivo*.

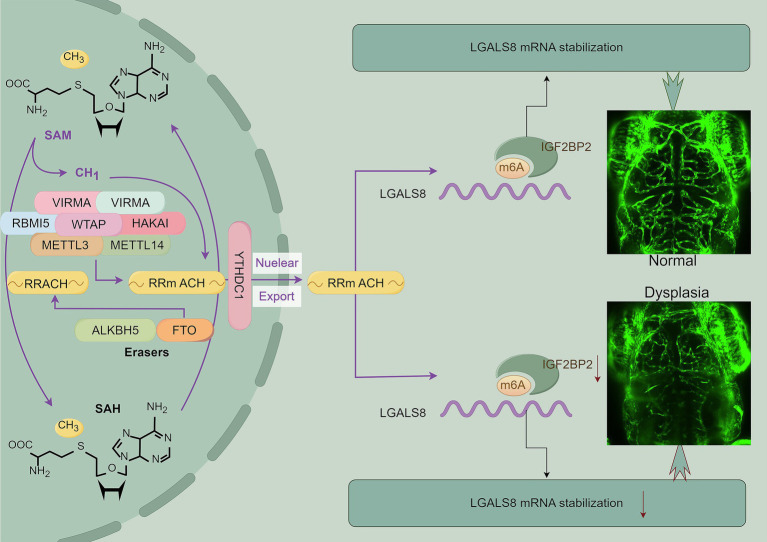

## Introduction

Cerebral arteriovenous malformations (AVMs) are anomalous tangles of blood vessels in the brain that disrupt normal blood flow. These malformations comprise intricate networks of arteries and veins interconnected by abnormal direct shunts, bypassing the capillary bed (1). This results in a high-pressure, high-flow vascular lesion that can lead to various neurological symptoms and potentially life-threatening complications, such as hemorrhage, seizures, and neurological deficits depending on their size, location, and the presence of associated complications (2). The management of cerebral AVMs often involves a multidisciplinary approach, including neurosurgery, endovascular therapy, and radiation therapy, depending on the specific characteristics of the AVM and the patient’s clinical presentation, aiming to prevent hemorrhage, alleviate symptoms, and reduce the risk of neurological deficits (3). However, in cases where malformed vessels extensively affect eloquent cerebral regions, the efficacy and safety of such invasive treatments may be inadequate (3). Consequently, the development of novel treatment strategies, particularly pharmacologic therapies, has emerged as an urgent clinical need. However, the exact cause of cerebral AVMs is not fully understood.

Epigenetic mechanisms, including DNA modification, histone modification, and chromatin remodeling, are reversible, heritable, playing critical roles in regulating various physiological and pathological processes without altering the DNA sequence. Epigenetic modifications can bridge the external environment and the vascular microenvironment, influencing the occurrence, development, and prognosis of cerebral AVMs by regulating related signaling pathways (4). Recent studies have shown that RNA modifications, in addition to those occurring in DNA and histones, also regulate biological processes. Over 170 different types of post-transcriptional RNA modifications have been identified across organisms ranging from archaea and bacteria to eukaryotes (5). RNA methylations regulate nearly all aspects of RNA processing, including splicing, nuclear export, stability, decay, and translation, thereby shaping the transcriptomic landscape (6, 7).

N^6^-methyladenosine (m^6^A), catalyzed by the core methyltransferase complex consisting of the catalytic subunits METTL3 and METTL14, along with the regulatory subunits WTAP and KIAA1429, is the most common and abundant RNA modification in eukaryotes (8–13). Recent evidence suggests that the brain has the highest abundance of m^6^A RNA methylation among all organs, indicating its regulatory role in central nervous system development and cerebrovascular remodeling (14). Dysregulation of METTL3 and WTAP has been observed in cerebral AVMs, impacting blood vessel formation through m^6^A modification (15–17). IGF2BPs (insulin-like growth factor 2 mRNA-binding protein family) act as readers for m^6^A modification by specifically binding to m^6^A-modified regions within target mRNAs, enhancing their stability and preventing degradation (18). Dysregulation of IGF2BP2 activity or expression disrupts the balance of m^6^A-modified transcripts, leading to aberrant gene expression and contributing to diseases such as diabetic vascular complications and vasculogenic mimicry (19, 20). However, its role in pathologic angiogenesis remains unclear.

In this study, we analyzed RNA-seq data of AVMs and found that downregulated genes were enriched in RNA metabolism-related pathways, including RNA metabolism and RNA biosynthetic processes. Among these genes, IGF2BP2 exhibited the greatest variation in expression levels, but its function in AVMs remains unclear. We discovered that IGF2BP2 deficiency impaired tube formation in endothelial cells and disrupted vascular development in zebrafish models. An integrated analysis of multi-omics data indicated that IGF2BP2 as an m^6^A reader involved in regulating LGALS8 mRNA stability in endothelial cells. Additionally, LGALS8 deficiency resulted in abnormal angiogenesis *in vitro* and led to vascular dysplasia in the zebrafish model. Thus, we propose that IGF2BP2 regulates LGALS8 expression in an m^6^A-dependent manner, leading to vascular malformation in cerebral AVMs. These findings provided a novel insight into the molecular mechanisms of cerebral AVM progression and presented a potential treatment strategy for cerebral AVMs.

## Materials and methods

### Gene silencing and overexpression

IGF2BP2 and LGALS8 were stably overexpressed in Human umbilical vein endothelial cells (HUVECs) using Lenti-oeIGF2BP2 and Lenti-oeLGALS8, respectively, constructed by Shanghai GeneChem Co., Ltd.; and knockdown of IGF2BP2 was achieved using Lv-shIGF2BP2 constructed by Shanghai GeneChem Co., Ltd. (Supplementary Table S1). Transient knockdown of LGALS8 in HUVECs was performed with siRNAs (Supplementary Table S1), synthesized by Shanghai GenePharma Co., Ltd., following the manufacturer’s protocol for Lipofectamine 3000 (Invitrogen). After 72 h of infection or 48 h of transfection, cells were harvested for subsequent mRNA or protein expression analysis.

### Quantitative reverse transcription polymerase chain reaction

Total RNA was extracted from cells using the Total RNA Extraction Kit (Solarbio). RNA was reverse transcribed into cDNA using the NovoScript^®^ Plus All-in-One 1st Strand cDNA Synthesis SuperMix (gDNA Purge) (Novoprotein E047-01B) according to the manufacturer’s instructions. Quantitative PCR amplification was then performed using the NovoStart^®^ SYBR qPCR SuperMix Plus (Novoprotein E096-01A). Relative quantification was conducted using the 2^−ΔΔCT^ method. All primer sequences are compiled in Supplementary Table S2.

### Western blot

Proteins were extracted from cells using lysis buffers containing detergents and protease inhibitors to prevent degradation. The protein concentration was determined using a BCA assay to ensure equal sample loading. Proteins were separated by molecular weight using SDS-PAGE (sodium dodecyl sulfate-polyacrylamide gel electrophoresis). Following electrophoresis, the separated proteins were transferred to a polyvinylidene difluoride (PVDF) membrane. The membrane was incubated in a blocking solution to prevent non-specific antibody binding. It was then incubated with a primary antibody specific to the target protein (IGF2BP2, abcam, ab124930, 1: 2000; LGALS8, abcam, ab109519, 1:1000; *β*-Actin, proteintech, 66009-1-Ig, 1:5000). After washing to remove unbound primary antibody, the membrane was incubated with a horseradish peroxidase (HRP)-conjugated secondary antibody (proteintech, SA00001-1, 1:5000; proteintech, SA00001-2, 1:5000). The chemiluminescence signal was visualized using the digital imaging systems.

### RNA sequencing and data analysis

RNA sequencing (RNA-seq) was conducted by Novogene. Briefly, RNA integrity was assessed using the RNA Nano 6000 Assay Kit of the Bioanalyzer 2100 system (Agilent Technologies, CA, United States). mRNA was purified using poly-T oligo-attached magnetic beads. Fragmentation was carried out using divalent cations under elevated temperature in First Strand Synthesis Reaction Buffer. First strand cDNA was synthesized using random hexamer primers and M-MuLV Reverse Transcriptase. Second strand cDNA synthesis was subsequently performed using DNA Polymerase I and RNase H. Sequencing adapters were then ligated to the ends of the cDNA fragments. PCR was performed with Phusion High-Fidelity DNA Polymerase, and the products were purified and assessed for quality on the Agilent Bioanalyzer 2100 system. Finally, the library preparations were sequenced on an Illumina Novaseq platform.

Raw sequencing data were subjected to quality control checks to remove low-quality reads and adapter sequences. High-quality reads were aligned to the reference genome using HISAT2. The abundance of transcripts was quantified by counting the number of reads mapped to each gene or transcript using featureCounts. FPKM (Fragments Per Kilobase of transcript per Million mapped reads) of each gene was calculated based on the length of the gene and the read count mapped to it. Differential expression analysis was performed using DESeq2 to compare gene expression levels between different conditions or treatments. The resulting *p*-values were adjusted using the Benjamini and Hochberg’s approach for controlling the false discovery rate. Genes with an adjusted *p*-value ≤ 0.05 identified by DESeq2 were considered differentially expressed. Gene enrichment analysis was conducted using Metascape (21).

### mRNA stability assays

Endothelial cells were cultured under appropriate conditions until they reached the desired confluency. Transcription was inhibited using actinomycin D, which prevents new mRNA synthesis. Endothelial cells were harvested at various time points after transcription inhibition (e.g., 0, 3, 6, and 9 h) to assess mRNA decay over time. Total RNA was extracted from each sample using the Total RNA Extraction Kit (Solarbio) to ensure high RNA integrity and purity. The extracted RNA was reverse transcribed into cDNA and quantified using qRT-PCR with gene-specific primers. The decay rate and half-life of the target mRNA were calculated based on the decrease in mRNA levels over time.

### Tube formation

Matrigel was pipetted into pre-chilled *μ*-Slide 15 Well 3D plates and allowed to polymerize at 37°C for 30 min. HUVECs were harvested, counted, and resuspended in an appropriate culture medium. The cells were then seeded onto the polymerized Matrigel at a density of 1–2 × 10 ^4^ cells per well. The plates were incubated at 37°C in a humidified atmosphere with 5% CO_2_. Tube formation was monitored at 24 h using an inverted microscope. Images of the formed tubular structures were captured for subsequent analysis. ImageJ software with the Angiogenesis Analyzer plugin was used to facilitate the analysis.

### Animals

AB wild-type and Tg (fli1:EGFP) strain zebrafish were all raised in fish farming water at 28°C (water quality: 200 mg instant sea salt was added to every 1 L of reverse osmosis water; conductivity was 450 ~ 550 μS/cm; pH was 6.5 ~ 8.5; hardness was 50 ~ 100 mg/L CaCO_3_). The feeding and management comply with the requirements of the international AAALAC certification (certification number: 001458). Zebrafish at the single-cell stage were used for gene target knockdown or rescue experiments. The Medical Research Ethics Committee of Zhengzhou Central Hospital approved all animal experiments (ZXYY2024129).

### Crispant and rescue zebrafish

AB wild-type strain zebrafish at the single-cell stage were randomly selected. A 1 nL mixture of gRNA (320 ng/μL, Supplementary Table S3) and Cas9 protein (800 ng/μL) was injected into each embryo. After 3 days of treatment at 28°C, 20 juvenile fish from each target site were randomly selected for genome extraction, PCR amplification, and sequencing. The PCR products were used to create TA clones, and 15 clones per target site were sequenced to calculate the target cleavage efficiency. Based on the cleavage efficiency and the presence of frameshift mutations, specific gRNAs were selected for subsequent experiments.

Tg (fli1:EGFP) strain zebrafish at the single-cell stage were randomly selected. The knockdown group was injected with a 1 nL mixture of gRNA (320 ng/μL) and Cas9 protein (800 ng/μL) per embryo, while the control group was injected with the same concentration of an ineffective target. For the rescue experiment, a 1 nL mixture of gRNA (320 ng/μL), Cas9 protein (800 ng/μL), overexpression plasmid (25 ng/μL, with DsRed fluorescent protein as a control), and TP mRNA (25 ng/μL) was injected per embryo. After the embryos were cultured at 28°C for 24 h, PTU was added to inhibit pigment growth. The 4 dpf zebrafish were fixed using 1.5% low-melting point agarose. Confocal imaging was performed using a 10X air microscope with a 2X zoom and an excitation wavelength of 488 nm.

### Datasets

RNA-seq data for control tissues and cerebral arteriovenous malformations were obtained from our previous study (16). MeRIP-seq and RNA-seq data for normal and METTL3 knockdown or WTAP knockdown HUVECs were obtained from the GEO database (GSE142386) (15, 16). RNA-seq data for normal and IGF2BP2 knockdown HUVECs generated in this study are available in the Supplementary Table S4.

### Statistical analysis

T-tests were used to analyze the significance of differences in gene expression, cell proliferation, and tube formation. All statistical analyses were performed using GraphPad Prism 9 and R software, with *p*-values less than 0.05 considered statistically significant.

## Results

### IGF2BP2 is downregulated in cerebral AVMs and regulates angiogenesis in endothelial cells

**Figure 1:**
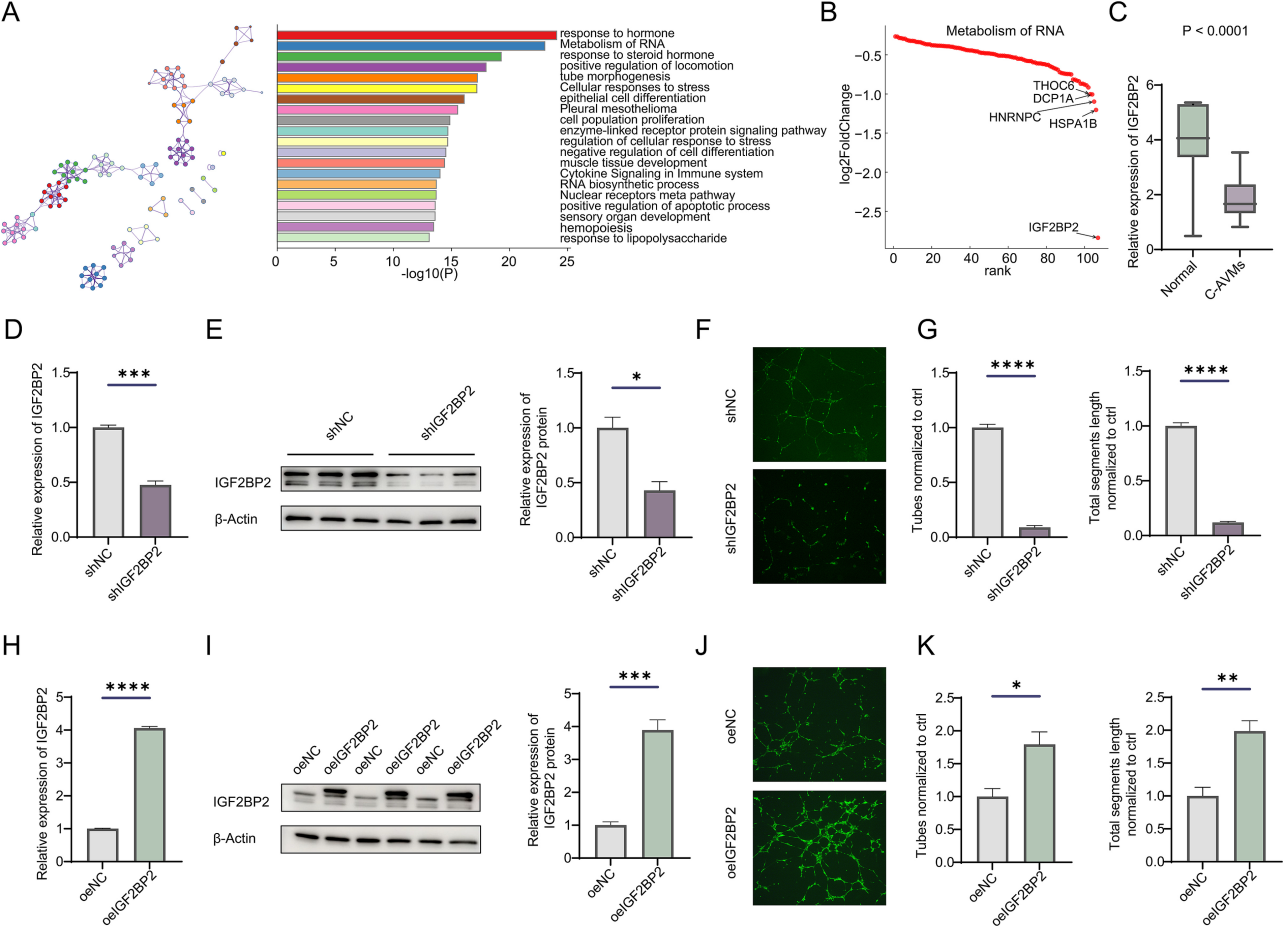
IGF2BP2 is downregulated in cerebral AVMs and regulates angiogenesis in endothelial cells. **(A)** Enrichment analysis of downregulated genes between normal tissues and cerebral arteriovenous malformations. **(B)** Rank plot showing genes enriched in the metabolism of RNA pathway. **(C)** Box plot showing the expression of IGF2BP2 between cerebral AVMs and normal tissues. **(D)** qRT-PCR and **(E)** Western blot detecting the IGF2BP2 knockdown efficiency in endothelial cells. **(F)** Representative images and **(G)** statistical analysis of tube formation assay in control and IGF2BP2-deficient endothelial cells. **(H)** qRT-PCR and **(I)** Western blotting detecting the IGF2BP2 overexpression in endothelial cells. **(J)** Representative images and **(K)** statistical analysis of tube formation assay in control and IGF2BP2-overexpressing endothelial cells. *p < 0.05; **p < 0.01; ***p < 0.001; ****p < 0.0001.

Previously, we analyzed transcriptional differences between cerebral arteriovenous malformations (AVMs) and normal tissues (Supplementary Figure S1A) (16). Enrichment analysis revealed that downregulated differentially expressed genes (DEGs) were significantly associated with pathways such as metabolism of RNA, RNA biosynthetic processes, epithelial cell differentiation, tube morphogenesis, and cellular responses to stress (Figure 1A; Supplementary Figure S1B). Given the regulatory role of RNA metabolism in vascular development, we further examined DEGs involved in RNA metabolic pathways and identified IGF2BP2 as the gene with the most pronounced expression difference (Figures 1B,C). However, the role of IGF2BP2 in AVM formation and development remains unclear. To determine its role in vascular development, we knocked down IGF2BP2 in endothelial cells (Figures 1D,E). Knockdown of IGF2BP2 significantly reduced the tube-forming ability of endothelial cells (Figures 1F,G), while overexpression of IGF2BP2 in endothelial cells promoted tube formation compared to controls (Figures 1H–K).

### IGF2BP2 regulates vascular development-related pathways in endothelial cells

**Figure 2:**
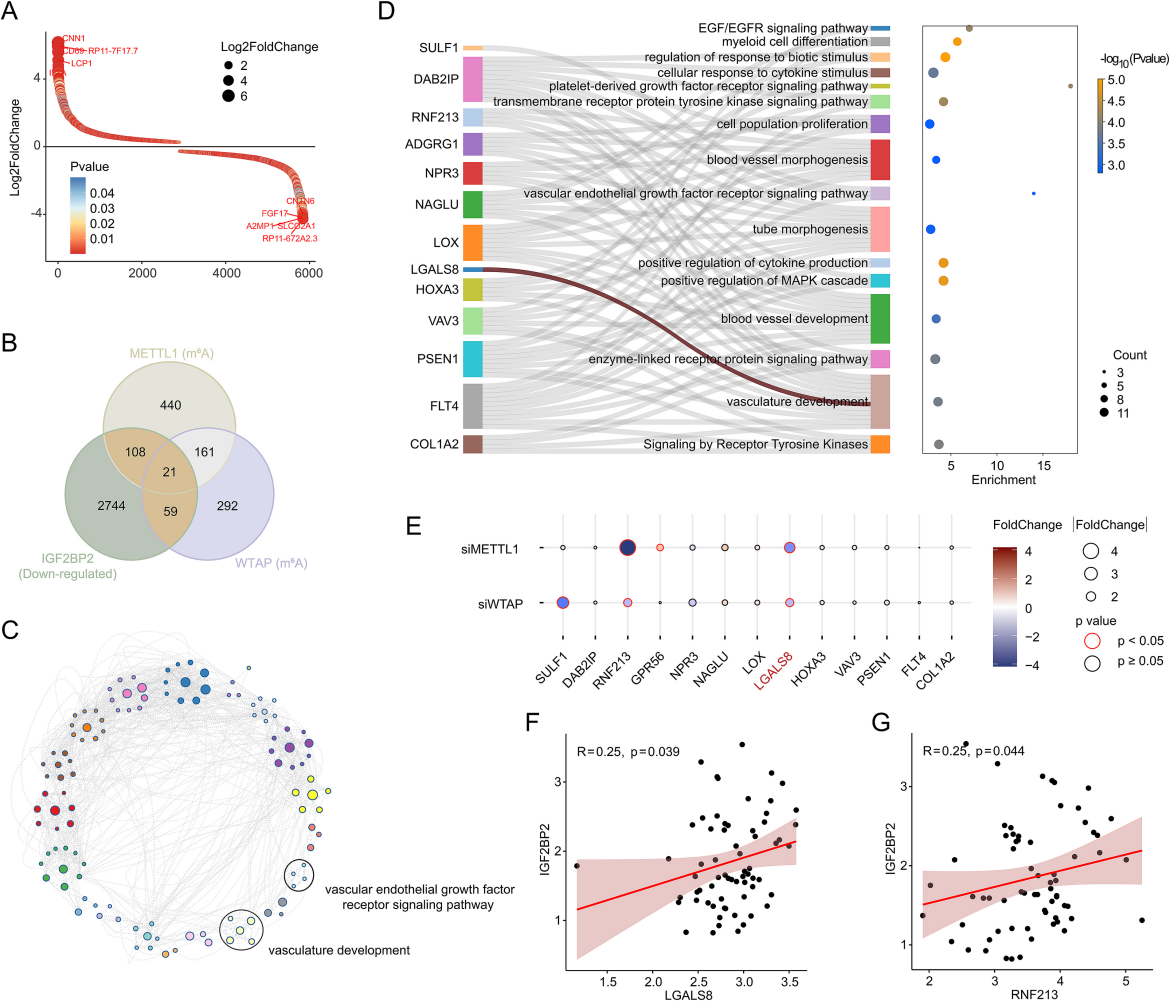
IGF2BP2 regulates vascular development-related pathways in endothelial cells. **(A)** Rank plot depicting differentially expressed genes between IGF2BP2 knockdown and control endothelial cells. **(B)** Venn diagram showing the intersection between downregulated genes in IGF2BP2 knockdown endothelial cells and genes downregulated at the level of m^6^A modification in METTL3-deficient or WTAP-deficient endothelial cells. **(C)** Network diagram displaying the results of enrichment analysis of target genes. **(D)** Sankey diagram showing the genes enriched in the vascular development-related pathways. **(E)** Bubble diagram displaying the expression levels between control and METTL3-deficient or WTAP-deficient endothelial cells. **(F)** Correlation analysis of IGF2BP2 expression with LGALS8 expression in cerebral AVMs. **(G)** Correlation analysis of IGF2BP2 expression with RNF213 expression in cerebral AVMs.

Next, we performed transcriptome sequencing of IGF2BP2 knockdown and control endothelial cells to determine how IGF2BP2 regulates vascular development. RNA-seq analysis identified 5,802 DEGs, including 2,870 upregulated and 2,932 downregulated DEGs (Figure 2A; Supplementary Figure S2). As IGF2BP2 acts as a reader for m^6^A modification, enhancing the stability of m^6^A-modified transcripts and preventing their degradation, we analyzed whether the m^6^A modification of downregulated genes in IGF2BP2-deficient endothelial cells was regulated by the N^6^-methyladenosine methyltransferase complex. By integrating MeRIP-seq data of control and METTL3-deficient or WTAP-deficient endothelial cells, we identified 188 target genes (Figure 2B). Enrichment analysis showed that these genes were mainly involved in the vascular endothelial growth factor receptor signaling pathway and vasculature development (Figure 2C), including SULF1, DAB2IP, RNF213, ADGRG1, NPR3, NAGLU, LOX, LGALS8, HOXA3, VAV3, PSEN1, FLT4, and COL1A2 (Figure 2D). We further analyzed whether the expression of the 13 genes enriched in vascular-related pathways was influenced by METTL3 and WTAP. RNA-seq results indicated that RNF213 and LGALS8 were downregulated in both METTL3-deficient and WTAP-deficient endothelial cells, while GPR56 and SULF1 were downregulated in METTL3-deficient and WTAP-deficient endothelial cells, respectively (Figure 2E). In addition, IGF2BP2 expression was significantly correlated with the expression of RNF213 and LGALS8 in cerebral AVMs (Figures 2F,G).

### IGF2BP2 regulates LGALS8 expression in endothelial cells

**Figure 3:**
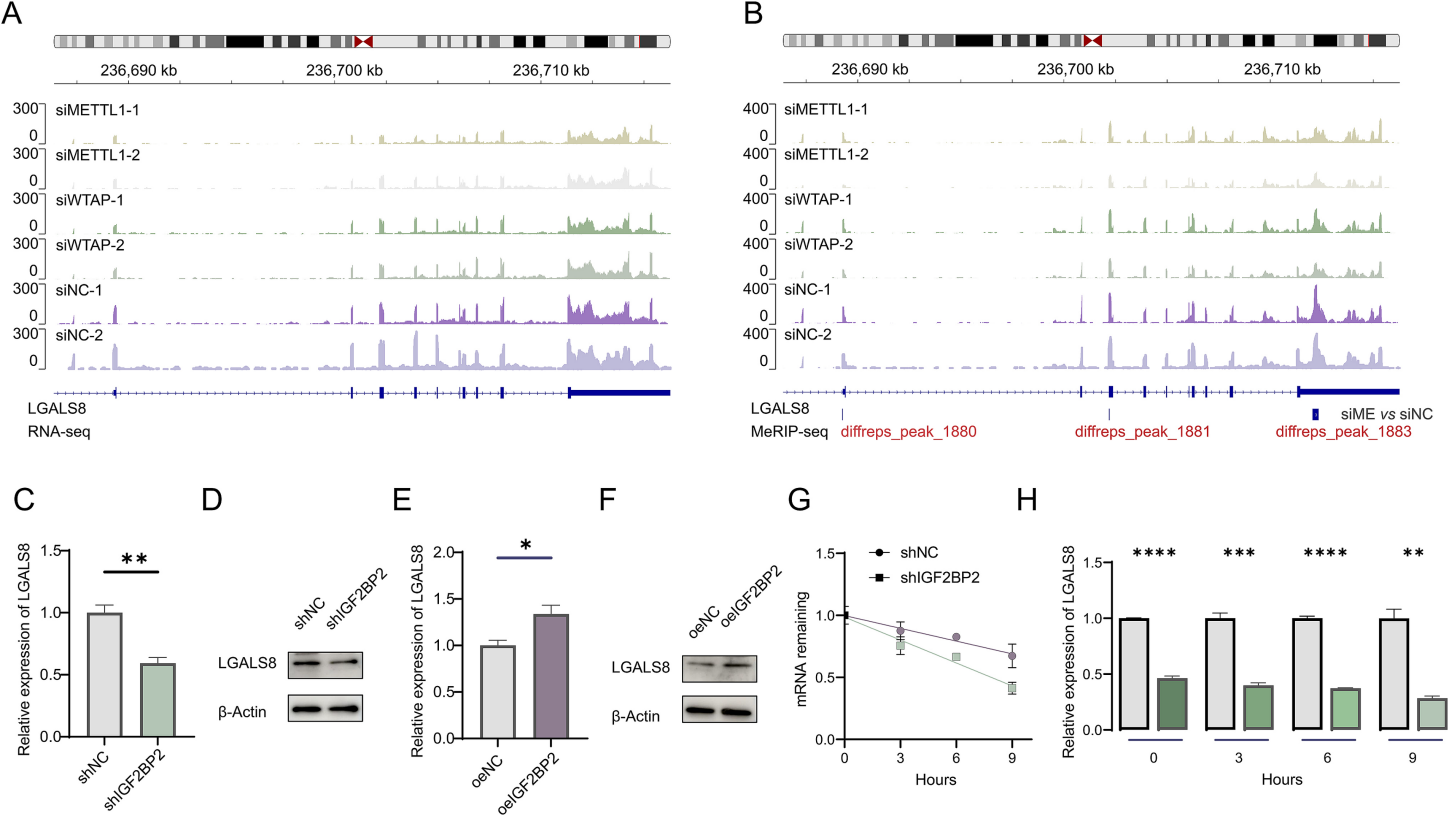
IGF2BP2 regulates LGALS8 expression in endothelial cells. **(A)** Integrative Genomics Viewer (IGV) tracks displaying RNA-seq reads distribution in LGALS8 mRNA. **(B)** IGV tracks displaying MeRIP-seq reads distribution in LGALS8 mRNA. **(C)** qRT-PCR detecting differences in LGALS8 expression between control and IGF2BP2 knockdown endothelial cells. **(D)** Western blot detecting differences in LGALS8 expression between control and IGF2BP2 knockdown endothelial cells. **(E)** qRT-PCR detecting differences in LGALS8 expression between control and IGF2BP2-overexpressing endothelial cells. **(F)** Western blot detecting differences in LGALS8 expression between control and IGF2BP2-overexpressing endothelial cells. **(G)** The mRNA half-life of LGALS8 transcript in control and IGF2BP2-overexpressing endothelial cells. **(H)** qRT-PCR detecting differences in LGALS8 expression between control and IGF2BP2 knockdown endothelial cells at specific time points. *p < 0.05; **p < 0.01; ***p < 0.001; ****p < 0.0001.

A comprehensive analysis of expression levels and m^6^A modifications suggested that IGF2BP2 may regulate LGALS8 in an m^6^A-dependent manner, thereby influencing vascular development (Figures 3A,B). To investigate this hypothesis, we examined whether IGF2BP2 regulates LGALS8 expression in endothelial cells. Compared to the control, LGALS8 expression was significantly reduced in IGF2BP2 knockdown endothelial cells (Figures 3C,D). Conversely, LGALS8 expression was significantly increased in IGF2BP2-overexpressing endothelial cells (Figures 3E,F). Since, IGF2BP2 enhances the stability of m^6^A-modified transcripts, we analyzed the mRNA half-life of the LGALS8 transcript in both control and IGF2BP2 knockdown endothelial cells. The results indicated that LGALS8 mRNA levels consistently decreased in IGF2BP2 knockdown endothelial cells compared to control endothelial cells at various time points following actinomycin D treatment, and the mRNA half-life of the LGALS8 transcript was shorter in IGF2BP2 knockdown endothelial cells compared to control endothelial cells (Figures 3G,H).

### LGALS8 deficiency inhibits angiogenesis in endothelial cells

**Figure 4:**
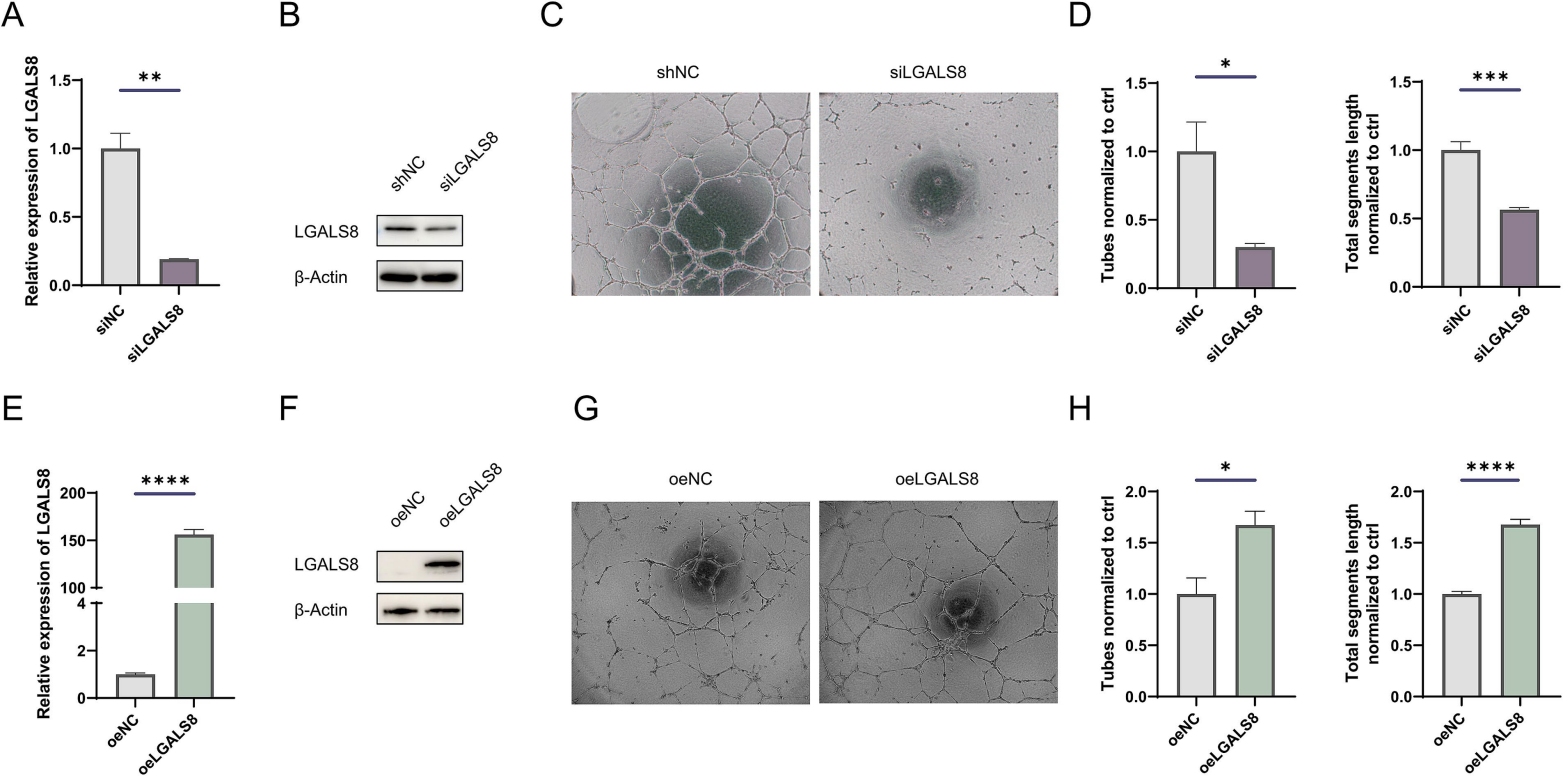
LGALS8 deficiency inhibits tube formation of endothelial cells. **(A)** qRT-PCR and **(B)** Western blot detecting the efficiency of LGALS8 knockdown in endothelial cells. **(C)** Representative images and **(D)** statistical analysis of tube formation assay in control and LGALS8-deficient endothelial cells. **(E)** qRT-PCR and **(F)** Western blotting detecting LGALS8 overexpression in endothelial cells. **(G)** Representative images and **(H)** statistical analysis of tube formation assay in control and LGALS8-overexpressing endothelial cells. *p < 0.05; **p < 0.01; ***p < 0.001; ****p < 0.0001.

To determine the role of LGALS8 in angiogenesis, we knocked down its expression in endothelial cells (Figures 4A,B). The results of the tube formation assay showed that LGALS8 knockdown significantly reduced the tube-forming ability of endothelial cells (Figures 4C,D). Additionally, we also overexpressed LGALS8 in endothelial cells (Figures 4E,F). The results of the tube formation assay indicated that overexpression of LGALS8 promoted tube formation compared to control endothelial cells (Figures 4G,H).

### IGF2BP2 and LGALS8 regulate vascular development in the zebrafish model

**Figure 5:**
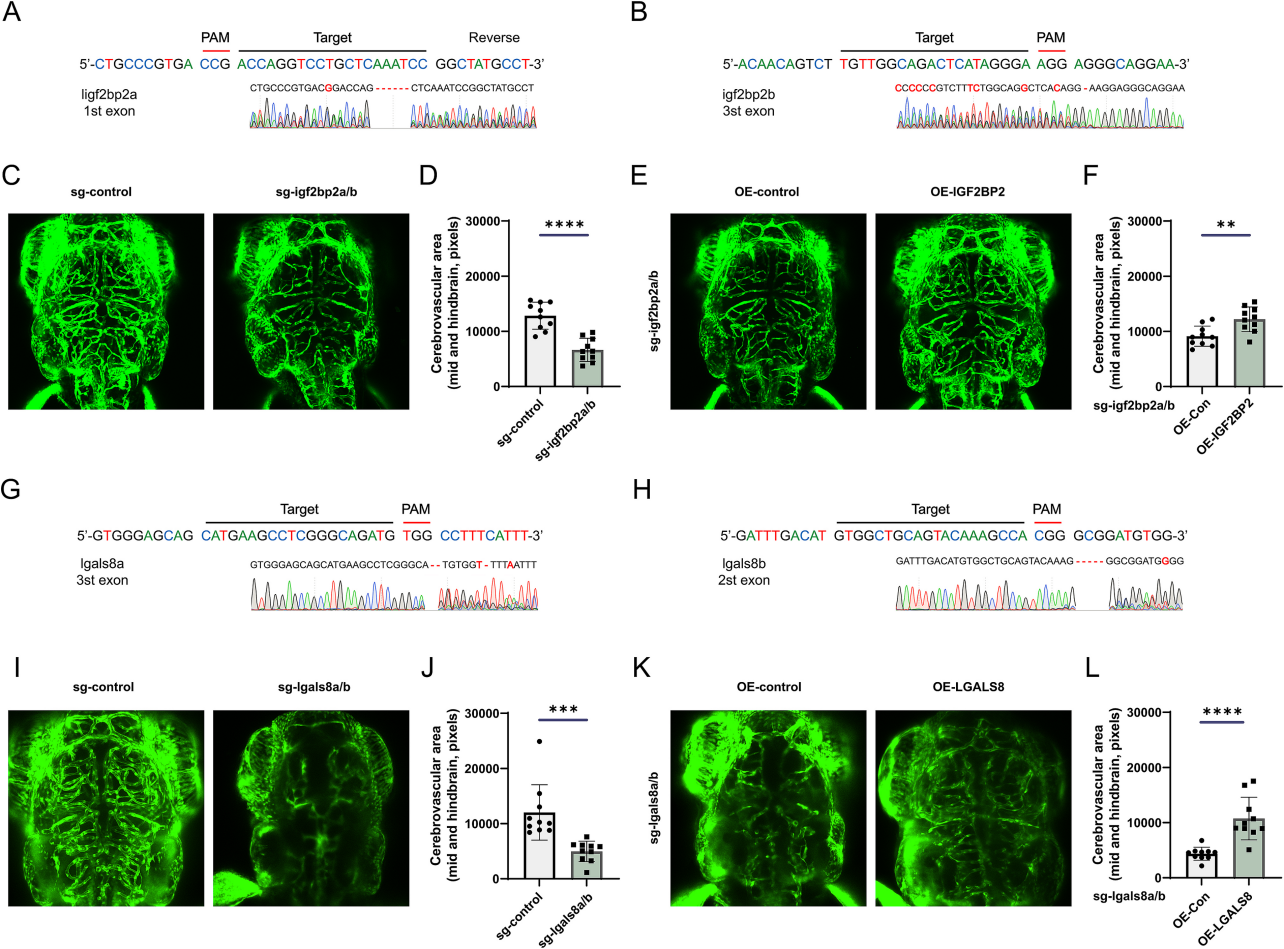
IGF2BP2 and LGALS8 regulate vascular development in the zebrafish model. **(A,B)** Schematic of CRISPR/Cas9-mediated generation of igf2bp2a/b crispant zebrafish and identification via RNA-sequencing. **(C)** Representative images of cerebrovascular structures and **(D)** statistical analysis of mid- and hind-brain vascular areas in control and igf2bp2a/b crispant zebrafish. **(E)** Representative images of cerebrovascular structures and **(F)** statistical analysis of mid- and hind-brain vascular areas in igf2bp2a/b crispant and rescue zebrafish. **(G,H)** Schematic of CRISPR/Cas9-mediated generation of lgals8a/b crispant zebrafish and identification via RNA-sequencing. **(I)** Representative images of cerebrovascular structures and **(J)** statistical analysis of mid- and hind-brain vascular areas in control and lgals8a/b crispant zebrafish. **(K)** Representative images of cerebrovascular structures and **(L)** statistical analysis of mid- and hind-brain vascular areas in lgals8a/b crispant and rescue zebrafish. *p < 0.05; **p < 0.01; ***p < 0.001; ****p < 0.0001.

To examine the role of IGF2BP2 in vascular development, we generated igf2bp2a/b crispant zebrafish using CRISPR-Cas9 (Figures 5A,B). Compared to control Tg (fli1:EGFP) zebrafish, the density of brain vessels was reduced in the igf2bp2a/b crispant zebrafish (Figures 5C,D). Additionally, we constructed rescue zebrafish by injecting an overexpression plasmid together with gRNA and Cas9 protein into embryos. Imaging results showed that the density of brain vessels in the IGF2BP2 rescue zebrafish was significantly increased compared to control zebrafish injected with DsRed fluorescent protein (Figures 5E,F). Similarly, we investigated the role of LGALS8 in vascular development (Figures 5G,H). We found that the density of brain vessels was dramatically reduced in the lgals8a/b crispant zebrafish (Figures 5I,J), and partially restored in the LGALS8 rescue zebrafish (Figures 5K,L).

## Discussion

Cerebral arteriovenous malformations are high-flow vascular lesions characterized by direct artery-to-vein connections without an intervening capillary network (1). This anatomical anomaly allows arterial blood to flow directly into the venous system with minimal resistance, significantly increasing the risk of catastrophic intracranial hemorrhage (22, 23). Cerebral AVMs account for approximately 50% of all pediatric intracerebral hemorrhage (ICH) and approximately 5–6% of adult ICH cases (24, 25). Unruptured cerebral AVMs have an annual hemorrhage rate of 1–3% (26–28), with the risk of rupture escalating to 5% following an initial hemorrhage (29). However, the processes behind cerebral AVMs genesis, progression, and development are still not well understood.

The dysregulation of immune system and increased the inflammatory reaction triggered by proinflammatory cytokines, neutrophils, macrophages, and so on, to incite injury, which in turn exacerbates the inflammation, recruits leukocytes, activates the endothelial cells of the AVM (30), and involves in changing the angioarchitectural structure of AVM through the upregulation of angiogenic factors (31). As a result, increased vascular damage resulting in AVM formation and expansion. In this study, we found that IGF2BP2 was downregulated in cerebral AVMs, and its deficiency in endothelial cells significantly enhanced the immunity activity, inflammatory response, and cytokine production (Supplementary Figure S2), indicating its role in injury of vascular and remodeling of angioarchitectural structure, which has been confirmed in the crispant zebrafish model (Figure 5).

IGF2BP2 enhances the stability of m^6^A-modified transcripts, preventing their degradation and prolonging their presence in the cytoplasm. Integrated analysis of multi-omics data revealed that differentially expressed genes regulated by IGF2BP2 dependent on m^6^A modification were mainly involved in immunity, vascular endothelial growth factor receptor signaling pathway and vasculature development (Figure 2C). A comprehensive analysis of expression and m^6^A modification levels suggested that LGALS8 was an important target regulated by IGF2BP2 in an m^6^A-dependent manner. LGALS8 encodes galectin-8, a member of the galectin family, which is involved in various biological processes, such as the development, angiogenesis, cell differentiation, adhesion, autophagy, immune response, and inflammation (32). It has been discovered that galectin-8 has important functions in the central nervous system, such as protecting neurons from harmful conditions such as ischemia and neurodegenerative diseases (33). Here, we found cerebrovascular dysplasia in the lgals8a/b crispant zebrafish model, demonstrating a role for LGALS8 in neurological vascular development (Figure 5). It has been reported that the N-terminal CRD of galectin-8 mediates the preferential binding of galectin-8 to farnesylated K-Ras4B, which consequently inhibits RAS activation (34). These findings suggest that galectin-8 deficiency may lead to KRAS activation, and hyperactivation of oncogenic KRAS has been reported to induce cerebral AVMs in mice (35). However, how LGALS8 deficiency leads to cerebrovascular dysplasia deserves further investigation.

## Conclusion

We found that IGF2BP2 was downregulated in cerebral AVMs and inhibited LGALS8 expression in an m^6^A-dependent manner, leading to cerebrovascular dysplasia *in vivo* (see Graphical abstract). These findings provided a novel insight into the molecular mechanisms of cerebral AVM progression and presented a potential treatment strategy for cerebral AVMs.

## Data Availability

The raw data supporting the conclusions of this article will be made available by the authors, without undue reservation.

## Supplementary material

Figure S1 Identification and enrichment analysis of differentially expressed genes between cerebral arteriovenous malformations and normal tissues. **(A)** Rank plot showing transcriptional differences between cerebral arteriovenous malformations and normal tissues. **(B)** Enrichment analysis of upregulated genes between normal tissues and cerebral arteriovenous malformations.

Figure S2 Enrichment analysis of differentially expressed genes between IGF2BP2 knockdown and control endothelial cells. **(A)** Enrichment analysis of differentially expressed genes between IGF2BP2 knockdown and control endothelial cells. **(B)** Enrichment analysis of differentially expressed genes between IGF2BP2 knockdown and control endothelial cells.
