# Supplementary figures and images for: Insulin like growth factor 2 mRNA binding protein 2 regulates vascular development in cerebral arteriovenous malformations
Source: Front Neurol. 2024 Dec 11;15:1483016. doi: 10.3389/fneur.2024.1483016 (PMC11668662; doi:10.3389/fneur.2024.1483016)

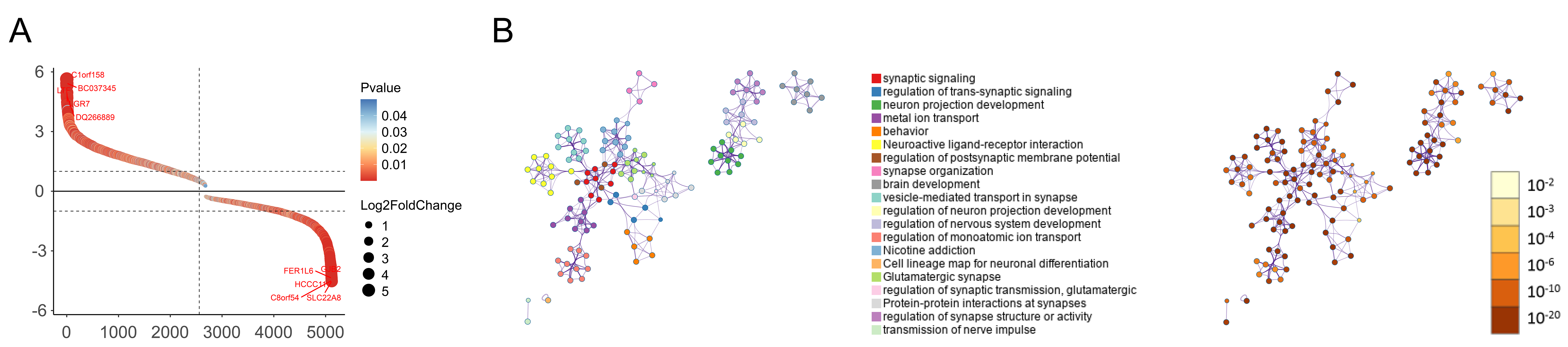

Supplement: Figure S1 — Identification and enrichment analysis of differentially expressed genes between cerebral arteriovenous malformations and normal tissues. (A) Rank plot showing transcriptional differences between cerebral arteriovenous malformations and normal tissues. (B) Enrichment analysis of upregulated genes between normal tissues and cerebral arteriovenous malformations. [file Image_1.TIF]

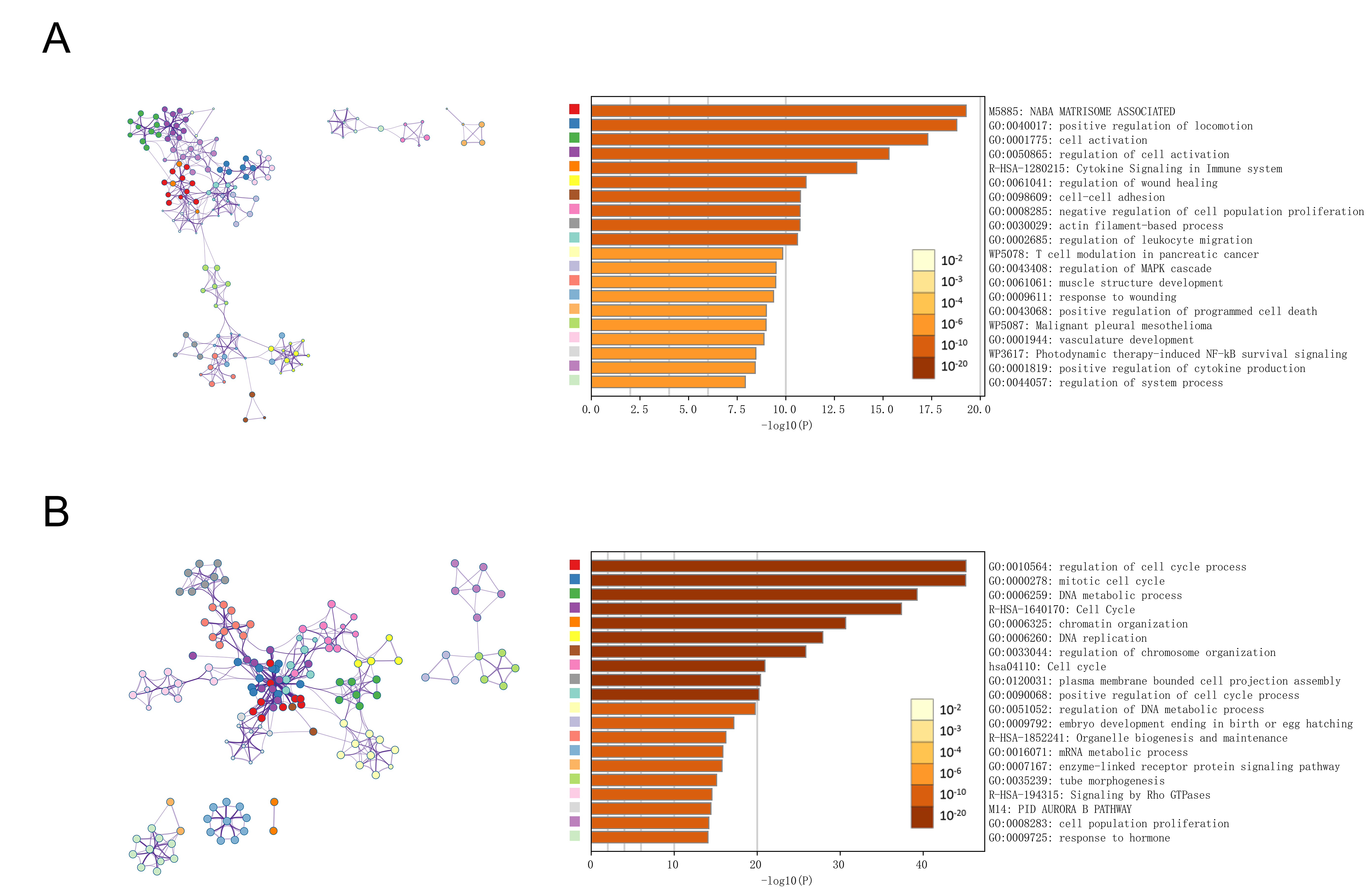

Supplement: Figure S2 — Enrichment analysis of differentially expressed genes between IGF2BP2 knockdown and control endothelial cells. (A) Enrichment analysis of differentially expressed genes between IGF2BP2 knockdown and control endothelial cells. (B) Enrichment analysis of differentially expressed genes between IGF2BP2 knockdown and control endothelial cells. [file Image_2.TIF]
